# *Morinda officinalis* oligosaccharides improves sperm motility via the gut microbiota and the IGF-1/PI3K/mTOR signaling pathway

**DOI:** 10.3389/fendo.2025.1711475

**Published:** 2026-01-05

**Authors:** Jiajia Hu, Guoxuan Li, Bingxue Ouyang, Anguo Wang, Yejuan Li, Hui Lu

**Affiliations:** 1 Reproductive Medical Center, Hainan Women and Children’s Medical Center, Haikou, Hainan, China; 2 Hainan Medical University–The University of Hong Kong Joint Laboratory of Tropical Infectious Diseases, Haikou, Hainan, China

**Keywords:** *Morinda officinalis* oligosaccharides, asthenozoospermia, sperm motility, gut microbiota, IGF-1/PI3K/mTOR signaling pathway

## Abstract

**Background:**

This study aimed to evaluate the efficacy of *Morinda officinalis* oligosaccharides (MOS) in improving sperm motility and quality in a gossypol-induced asthenozoospermia mouse model, and to explore the potential underlying mechanisms.

**Methods:**

Male C57BL/6J mice were randomly assigned to six groups: normal control, model, low-dose MOS (12.5 mg/kg), medium-dose MOS (25 mg/kg), high-dose MOS (50 mg/kg), and positive control (L-carnitine 10 mg/kg). Asthenozoospermia was induced by gossypol (20 mg/kg) injection every 3 days for 30 days, followed by MOS gavage for 14 days. Sperm motility, hormone levels, and histopathological changes were analyzed, and gut microbiota composition was analyzed using 16S rRNA sequencing. Mechanistic validation was performed using *Anaerococcus* transplantation and rapamycin co-treatment with MOS.

**Results:**

Gossypol impaired sperm concentration and motility and induced luminal sperm depletion with vacuolation in the epididymis, without overt structural lesions in the testes, liver, or kidneys. MOS improved sperm motility in a dose-dependent manner, restored testosterone, normalized white blood cells and blood urea nitrogen levels, and reduced sperm morphological abnormalities. High-dose MOS significantly increased microbial α-diversity and enriched *Anaerococcus*, shifted the microbial community structure toward that of the normal group. MOS and *Anaerococcus* transplantation activated the IGF-1/PI3K/mTOR pathway, increased Bcl-2, and reduced Cleaved Caspase-3 expression, whereas rapamycin attenuated these improvements, indicating pathway dependence.

**Conclusions:**

MOS ameliorates gossypol-induced asthenozoospermia by remodeling the gut microbiota and activating IGF-1/PI3K/mTOR signaling, thereby improving sperm motility and reducing apoptosis. These findings highlight MOS as a promising microbiota-modulating therapeutic strategy for male reproductive dysfunction.

## Introduction

Asthenozoospermia, characterized by reduced progressive motility of spermatozoa, is one of the leading causes of male infertility (1). It is estimated that 8%-12% of couples of reproductive age globally are affected by infertility, with nearly half attributable to male-related causes (2). Sperm motility is regulated by multiple factors, including flagellar structural integrity, mitochondrial energy supply, and the stability of the reproductive tract microenvironment (3). Previous studies have demonstrated that the pathogenesis of asthenozoospermia is highly complex, involving genetic mutations, epigenetic regulation, energy metabolism disorders, oxidative stress, and mitochondrial dysfunction (4, 5).

In recent years, the gut microbiota, which is often referred to as the “second genome,” has attracted increasing attention for its role in male reproductive health (2, 6). The concept of the “gut–testis axis” suggests that intestinal microecology regulates inflammatory responses, sex hormone metabolism, blood-testis barrier integrity, and testicular function through metabolites such as short-chain fatty acids (SCFAs), ultimately affecting spermatogenesis and sperm maturation (7, 8). Clinically, treatments such as antioxidant therapy, hormone modulators, and assisted reproductive technologies (e.g., intracytoplasmic sperm injection, ICSI) can partially improve fertility outcomes; however, they fail to fundamentally reverse sperm dysfunction (3, 9). Emerging evidence indicates that microecological interventions, including probiotic supplementation and fecal microbiota transplantation (FMT), may improve sperm motility and hormone levels in animal models, highlighting the “microbiota-metabolite-signaling axis” as a potential novel therapeutic target (10, 11).

Traditional Chinese medicine (TCM) has a long-standing history in the treatment of male infertility. Classic formulations, such as Guilingji and Jinkui Shenqi Pill, have been shown to enhance sperm quality by modulating sex hormone levels and improving testicular structure (12). *Morinda officinalis* How (commonly known as Bajitian), one of the authentic “southern herbal medicines” (Nanyao) from Lingnan, was first recorded in the Shennong Bencao Jing. Traditionally, it has been used to treat impotence, infertility, and musculoskeletal disorders by “reinforcing kidney-yang, strengthening tendons and bones, and dispelling wind-dampness” (13). Modern pharmacological studies have demonstrated that *Morinda officinalis* exhibits diverse bioactivities, including neuroendocrine modulation, antioxidative stress, and anti-osteoporotic effects, particularly in kidney-yang deficiency syndromes (14, 15). In TCM andrology, it is often combined with *Lycium barbarum* and *Ligustrum lucidum* in “kidney-tonifying and essence-replenishing” prescriptions to treat infertility associated with kidney deficiency (16). Network pharmacology and molecular docking studies suggest that the combination of *Morinda officinalis* and *Lycium barbarum L* activates the Phosphoinositide 3-Kinase (PI3K)/Protein Kinase B (Akt) signaling pathway, reduces oxidative stress, enhances reproductive function, and improves sperm count and motility (17).

In recent years, oligosaccharide constituents of *Morinda officinalis*, collectively referred to as *Morinda officinalis* oligosaccharides (MOS), have received increasing scientific interest. These include bajijiasu, fructooligosaccharides, and inulin-type oligosaccharides, known for their water solubility. MOS exert various pharmacological effects by modulating gut microbiota, enhancing immune function, and mitigating oxidative stress (18, 19). A study has indicated that MOS possess multiple regulatory activities across the nervous, immune, and reproductive systems, including antidepressant, pro-angiogenic, bone metabolism-modulating, and fertility-enhancing effects (18). However, the underlying mechanisms by which MOS ameliorates asthenozoospermia remain unclear.

This study aimed to systematically investigate the mechanistic role of MOS in an experimental model of asthenozoospermia, with particular focus on its capacity to modulate sperm function via the “microbiota-signaling axis.” The findings are expected to provide theoretical and practical insights for the modern translational application of TCM-derived agents and for elucidating the role of gut microecology in male reproductive health. Therefore, the present work integrates hormonal, histological, and microbial analyses to provide a multi-dimensional evaluation of MOS action on the gut–testis axis and its potential to improve sperm motility through signaling regulation.

## Materials and methods

### Experimental animals

Eight-week-old specific pathogen-free (SPF) male C57BL/6J mice (body weight 22 ± 2 g; Beijing Vital River Laboratory Animal Technology Co., Ltd.; license No. SCXK (Jing) 2022-0052) were used. Animals were housed at 22 ± 2°C with 55% ± 5% relative humidity under a 12 h light/dark cycle and allowed ad libitum access to food and water after a 7-day acclimatization. The protocol was approved by the Animal Ethics Committee of Hainan Medical University (Approval No. HYALL-2025-003). All procedures complied with the National Institutes of Health (NIH) guidelines and were reported in accordance with ARRIVE 2.0.

### Animal grouping and model establishment

Mice were randomly allocated (random number table) into six groups (*n* = 6 per group): (i) normal; (ii) model; (iii) low-dose MOS (12.5 mg/kg); (iv) medium-dose MOS (25 mg/kg); (v) high-dose MOS (50 mg/kg) (20) and (vi) positive control (L-carnitine, 10 mg/kg, intraperitoneal injection) (21). MOS was purchased from Beijing Tongrentang Company Limited (Lot No. 2021B03527; 0.3 g per capsule; MOS 150 mg per capsule). Asthenozoospermia was induced by intraperitoneal injection of gossypol (20 mg/kg; HY-13407, Medchemexpress) every 3 days for 30 days, as previously reported (22); controls received equal volumes of 0.9% NaCl. Beginning the day after modeling, the three MOS groups received MOS by oral gavage with a dosing volume of 0.1 mL/10 g once/day for 14 consecutive days; the model and normal groups received equal volumes of saline. Body weight was recorded every 6 days, and coat condition, activity, feeding, and responsiveness were monitored throughout.

### Hematology and hepatic and renal function assessment

At the end of treatment, mice were anesthetized with isoflurane and whole blood was collected via retro-orbital venous plexus puncture. For hematology, 75 μL of whole blood was transferred into EDTA-K2 microtubes; for biochemistry, 300 μL was placed into clot activator tubes, rested at room temperature for 30 min, and centrifuged at 3,000 × *g* for 10 min to obtain serum. A fully automated hematology analyzer (Mindray BC-2800) measured white blood cells (WBC). Serum alanine aminotransferase (ALT) (Abcam, ab285263) and blood urea nitrogen (BUN) (Abcam, ab83362) were determined using commercial colorimetric kits according to manufacturers’ instructions. Absorbance was read on a microplate reader (BioTek Epoch) at appropriate wavelengths (450–550 nm) and concentrations were calculated from standard curves to evaluate potential hepatic and renal effects.

### Serum hormone measurements

Following the manufacturers’ protocols for testosterone (T; MU30398, Bio-swamp, Wuhan, China), follicle-stimulating hormone (FSH; MU30265, Bio-swamp), and luteinizing hormone (LH; MU30382, Bio-swamp) ELISA kits, standards, serum samples, and diluents were sequentially added to 96-well plates, sealed, incubated, washed, and developed. Absorbance at 450 nm was recorded using a microplate reader (SpectraMax Plus384, Molecular Devices, Sunnyvale). Concentrations of T, FSH, and LH were calculated from standard curves.

### Hematoxylin and eosin staining and histopathology

After deep anesthesia with an intraperitoneal overdose of sodium pentobarbital (150 mg/kg; Sigma-Aldrich, P010500) and confirmed cessation of respiration and heartbeat, mice were dissected immediately. Liver, kidney, epididymis, and testis were excised, weighed, and fixed in 10% neutral-buffered formalin for 48 h, dehydrated in graded ethanol, cleared in xylene, embedded in paraffin, and sectioned at 4 μm. Sections were deparaffinized, rehydrated, stained with hematoxylin for 8 min, rinsed, differentiated in acid alcohol for 30 s, blued for 2 min, counterstained with eosin for 2 min, dehydrated, cleared, and mounted with neutral resin (Solarbio, G1120). Slides were examined and imaged under a light microscope (Olympus BX53).

### Sperm analysis

Epididymides were placed in pre-warmed Modified Human Tubal Fluid (mHTF; Irvine Scientific). Tissues were gently punctured with a sterile syringe needle to release sperm into the medium. Dishes were incubated at 37°C for 15 min to promote sperm dispersal. Sperm suspensions were then loaded into counting chambers and analyzed using a computer-assisted sperm analysis (CASA) system (Hamilton Thorne IVOS II) for sperm concentration, total motility, and progressive motility.

### Scanning electron microscope observation of sperm morphology

The collected sperm samples were immediately immersed in electron microscope fixative (G1102, Servicebio) and fixed at room temperature for 2 h, then transferred to 4°C for storage. After fixation, the samples were washed three times with 0.1M phosphate buffer (PB, pH 7.4) for 15 min each time. Subsequently, the samples were fixed in 1% osmium tetroxide solution (Ted Pella Inc.) prepared in 0.1M PB under light protection for 1–2 h. After osmium tetroxide fixation, the samples were washed three times with 0.1M PB for 15 min each time to remove residual reagents. The samples were then dehydrated through an ethanol gradient, sequentially placed in 30%, 50%, 70%, 80%, 90%, 95%, 100%, and 100% anhydrous ethanol (100092183, China National Pharmaceutical Group Corporation) for 15 min at each concentration, followed by replacement with isoamyl acetate (10003128, China National Pharmaceutical Group Corporation) for 15 min. Once dehydration was complete, the samples were processed using a critical point dryer, with liquid CO_2_ as the transition medium, and the temperature and pressure were gradually increased to transition the liquid to gas, avoiding structural damage from sudden pressure changes. After drying, the samples were mounted on conductive carbon film double-sided adhesive and gold-coated using an ion sputtering device (108Auto, Cressington) for approximately 30 s to achieve a uniform metal film layer of 10–15 nm thickness, enhancing conductivity while avoiding covering fine surface structures. The prepared samples were observed under a scanning electron microscope (FEI Quanta250, FEI Company, USA). Imaging was performed at an accelerating voltage of 3 kV. After panoramic scanning at low magnification, the image was gradually magnified to high magnification to capture structural details of the head and tail.

### 16S rRNA gene high-throughput sequencing

At the end of treatment, mouse feces were collected, and microbial DNA was extracted from the feces. Samples failing quality control at the stages of DNA extraction, library construction, or sequencing were excluded from subsequent microbiota analyses. The composition of the gut microbiota was analyzed by sequencing 16S rDNA amplicons using the Illumina MiSeq platform with 2 × 300 bp paired-end reads (23). Primers 341F (5’-CCTACGGGGNGGCWGCAG-3’) and 806R (5’-GGACTACHVGGGGTATCTAAT-3’) were selected to amplify the V3-V4 region with an amplification length of 470 bp, to generate amplicons, and for taxonomic analysis. PCR amplicons were purified, identified, and paired-end sequenced, and sequences showing ≥97% similarity were clustered into operational taxonomic units (OTUs) using QIIME 2. Each representative sequence within an OTU was classified, and the resulting data were sequentially spliced, filtered, clustered in OTUs, and computed to obtain information on the corresponding substances and their distribution. Functional prediction was performed using PICRUSt2 (v2.5.0) based on the OTU table to infer the metagenomic functional composition of the gut microbiota. Predicted KEGG pathways were analyzed and visualized using R software (v4.3.1). Sankey diagrams were generated with the ggalluvial package to illustrate the associations between bacterial genera and predicted KEGG functional pathways.

### Preparation of *Anaerococcus* strain solution

The bacterial strain used was *Anaerococcus prevotii* ATCC 9321 (= DSM 20548, type strain PC1T), obtained from ATCC. *Anaerococcus prevotii* is a Gram-positive, obligately anaerobic, non-motile coccus that grows optimally at 37°C. The strain was cultured under strictly anaerobic conditions (85% N_2_, 10% H_2_, 5% CO_2_) in Reinforced Clostridial Medium (RCM; Oxoid) supplemented with 0.05% sodium cysteine. Colonies were typically visible after 72 h at 37°C. Growth was monitored by OD_600_; cells harvested at OD_600_ 0.6-0.8 were washed twice and resuspended in sterile PBS to 1 × 10^9^ CFU/mL (titer verified by anaerobic plate counts with serial dilution).

### Strain transplantation and rapamycin intervention

Asthenozoospermia was induced in male C57BL/6J mice by gossypol via intraperitoneal injection as above. Mice were randomized into five groups (*n* = 5/group): (i) normal; (ii) model; (iii) MOS-H: high-dose MOS (50 mg/kg); (iv) *Anaerococcus* group: 0.2 mL/day bacterial suspension (1 × 10^9^ CFU/mL) for 7 days; (v) MOS + rapamycin: MOS plus intraperitoneal rapamycin (Sigma, V900930-1MG) 8 mg/kg daily for 7 days (24). Mice not receiving rapamycin were given equal volumes of vehicle intraperitoneally. Efficacy was assessed comprehensively by CASA-derived sperm motility parameters, sex hormones and expression of proteins related to the Insulin-like Growth Factor 1 (IGF)-1/PI3K/mTOR pathway.

### Western blot

Epididymal tissue samples were lysed on ice for 30 min in RIPA buffer containing protease and phosphatase inhibitors (Beyotime, P0013B) supplemented with 10 mM NaF and 1 mM Na_3_VO_4_ (Sigma, S7920 and S6508). Lysates were centrifuged at 12,000 × g, 4°C for 10 min; supernatants were quantified by BCA assay (Thermo, 23227) and denatured at 95°C for 5 min in 5× SDS loading buffer containing dithiothreitol (DTT, 100 mM). Equal protein (30 μg/lane) was separated on 10% SDS-PAGE and transferred to PVDF membranes (Millipore, IPVH00010). After blocking with 5% BSA at room temperature for 1 h, membranes were incubated overnight at 4°C with primary antibodies: Akt (Cell Signaling Technology, 9272, 1: 1,000), phosphorylated Akt (Cell Signaling Technology, 9271, 1: 1,000), IGF-1 (Cell Signaling Technology, 73034, 1: 1,000), PI3K (Cell Signaling Technology, 4257, 1: 1,000), phosphorylated PI3K (p-PI3K; Cell Signaling Technology, 17366, 1: 1,000), mTOR (Cell Signaling Technology, 2983, 1: 1,000), phosphorylated mTOR (p-mTOR; Cell Signaling Technology, 5536, 1: 1,000), Cleaved Caspase-3 (Cell Signaling Technology, 9661, 1: 1,000) and GAPDH (Abcam, ab128915, 1: 10,000). HRP-conjugated anti-rabbit secondary antibody (Cell Signaling Technology, 7074, 1: 1,000) was applied for 1 h at 37°C. Bands were visualized with ECL (Beyotime, P0018FS) and imaged on a ChemiDoc system (Bio-Rad). Densitometry was performed in ImageJ with GAPDH normalization.

### Statistical analysis

Data are presented as mean ± standard deviation (SD). Analyses were performed in GraphPad Prism 10.0 (GraphPad Software, San Diego, CA, USA) and R. Data were tested for normality using the Shapiro–Wilk test and for homogeneity of variance using Levene’s test. One-way analysis of variance (ANOVA) was used for multiple-group comparisons, followed by Dunnett’s *post hoc* test for pairwise contrasts. Multiple testing was controlled using the Benjamini-Hochberg procedure where applicable. *P* < 0.05 was considered statistically significant.

## Results

### MOS improves sperm motility parameters in asthenozoospermic mice and exhibit favorable safety

**Figure 1:**
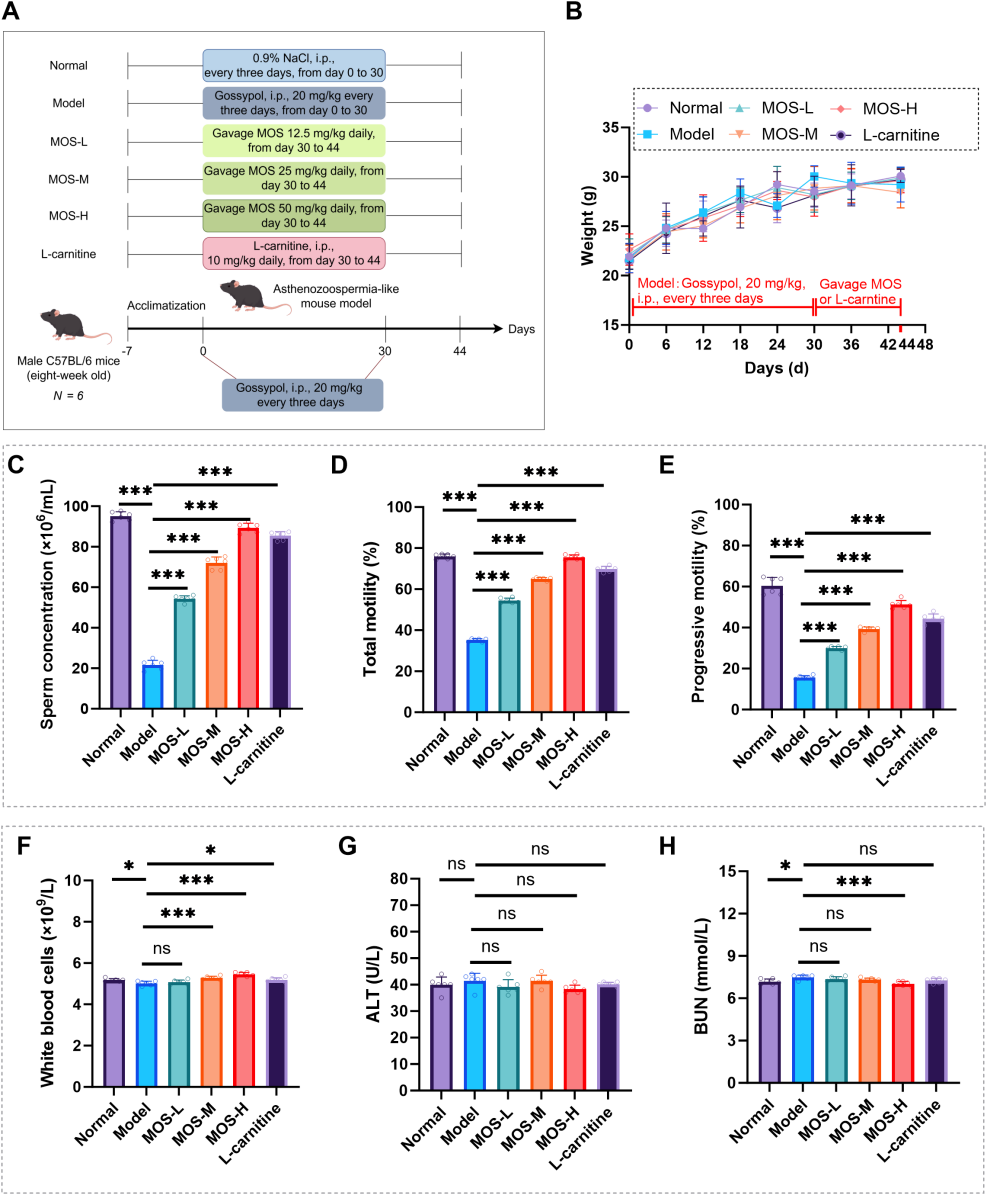
MOS intervention improves sperm quality and related physiological indicators in gossypol-induced asthenozoospermic mice. **(A)** Experimental grouping and dosing schedule. **(B)** Body weight changes of mice in different treatment groups (*n* = 6). **(C)** Sperm concentration (*n* = 6). **(D)** Total sperm motility (*n* = 6). **(E)** Progressive sperm motility (*n* = 6). **(F)** White blood cell count (*n* = 6). **(G)** Serum alanine aminotransferase (ALT) detection (*n* = 6). **(H)** Blood urea nitrogen (BUN) detection (*n* = 6). Data are presented as mean ± SD. Compared with the model group, ^*^P < 0.05, ^**^P < 0.01, ^***^P < 0.001, ns indicates no significant difference. MOS, *Morinda officinalis* oligosaccharides; ALT, Alanine aminotransferase; BUN, Blood urea nitrogen.

**Figure 2:**
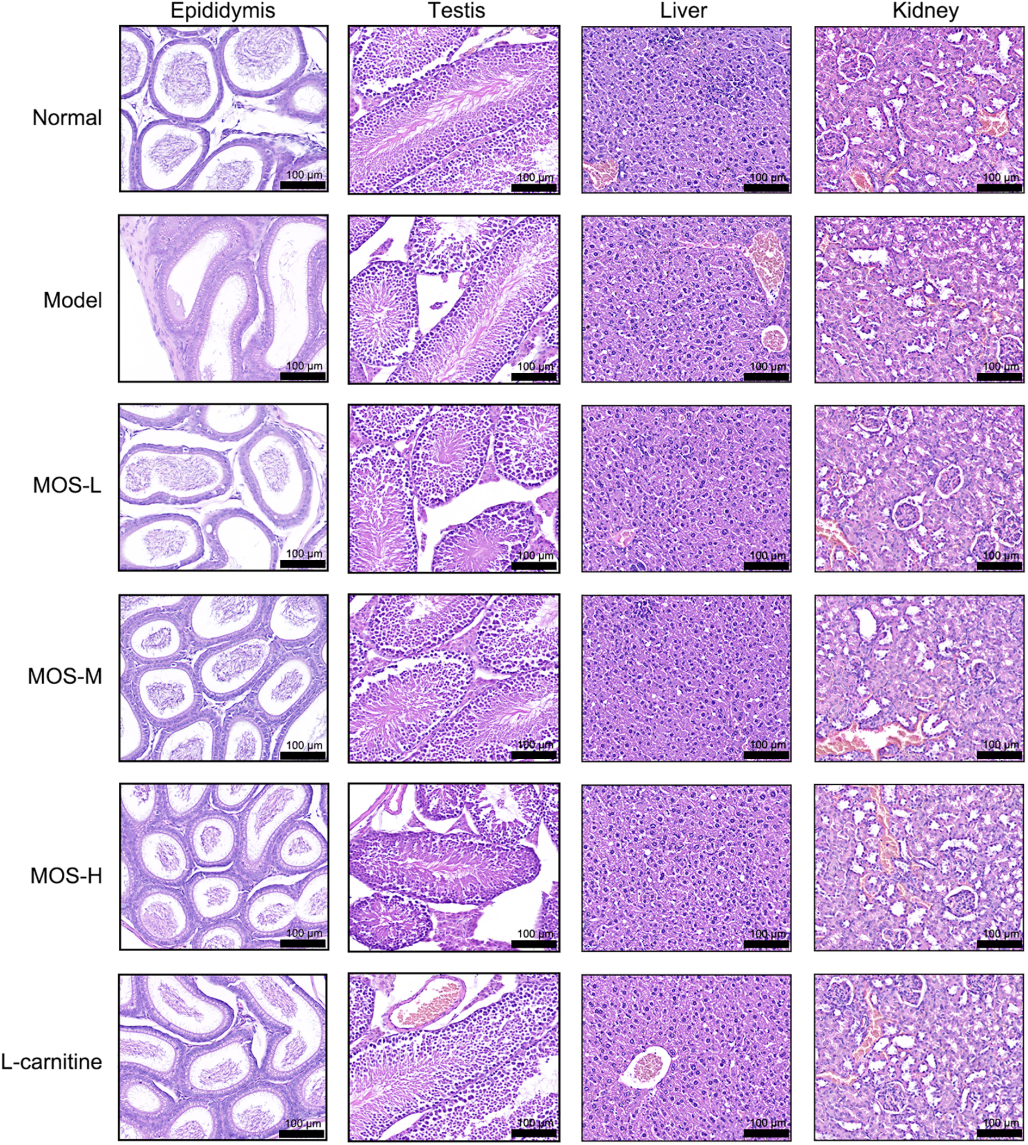
H&E staining of the testis, epididymis, liver, and kidney in different treatment groups (*n* = 6). Scale bar = 100 μm. MOS: *Morinda officinalis* oligosaccharides.

**Figure 1A** illustrates the experimental design and grouping strategy. During the intervention period, body weight increased steadily in all groups, and overall health status remained stable without abnormal changes (**Figure 1B**). After gossypol induction, compared with the Normal group, the Model group exhibited significant reductions in sperm concentration (**Figure 1C**), total motility (**Figure 1D**), and progressive motility (**Figure 1E**). MOS administration significantly improved these sperm parameters in a dose-dependent manner (**Figures 1C–E**). The positive control, L-carnitine, also showed the expected trend of improvement, while the Normal group maintained stable kinematic parameters within the physiological range. In addition, white blood cell (WBC) counts were significantly reduced in the Model group, whereas MOS treatment markedly restored WBC levels (**Figure 1F**). Although the WBC levels showed a statistically significant difference among groups, all values remained within the physiological range, suggesting limited biological relevance. Serum ALT levels showed no significant differences among groups (**Figure 1G**), whereas serum BUN was elevated in the Model group and significantly reduced by MOS treatment (**Figure 1H**). H&E staining revealed no overt structural lesions in the testes, liver, or kidneys. In contrast, the epididymis in the Model group exhibited reduced luminal sperm content accompanied by prominent luminal vacuolation, consistent with the significant decline in sperm concentration. Partial vacuolation was also observed in the MOS-L group, whereas these abnormalities were substantially ameliorated in the MOS-M, MOS-H, and L-carnitine groups (**Figure 2**). These findings indicate that MOS alleviates gossypol-induced sperm quality impairment in mice and is well tolerated within the tested dose range.

### MOS improves sex hormone levels and reduces sperm morphological abnormalities in asthenozoospermic mice

**Figure 3:**
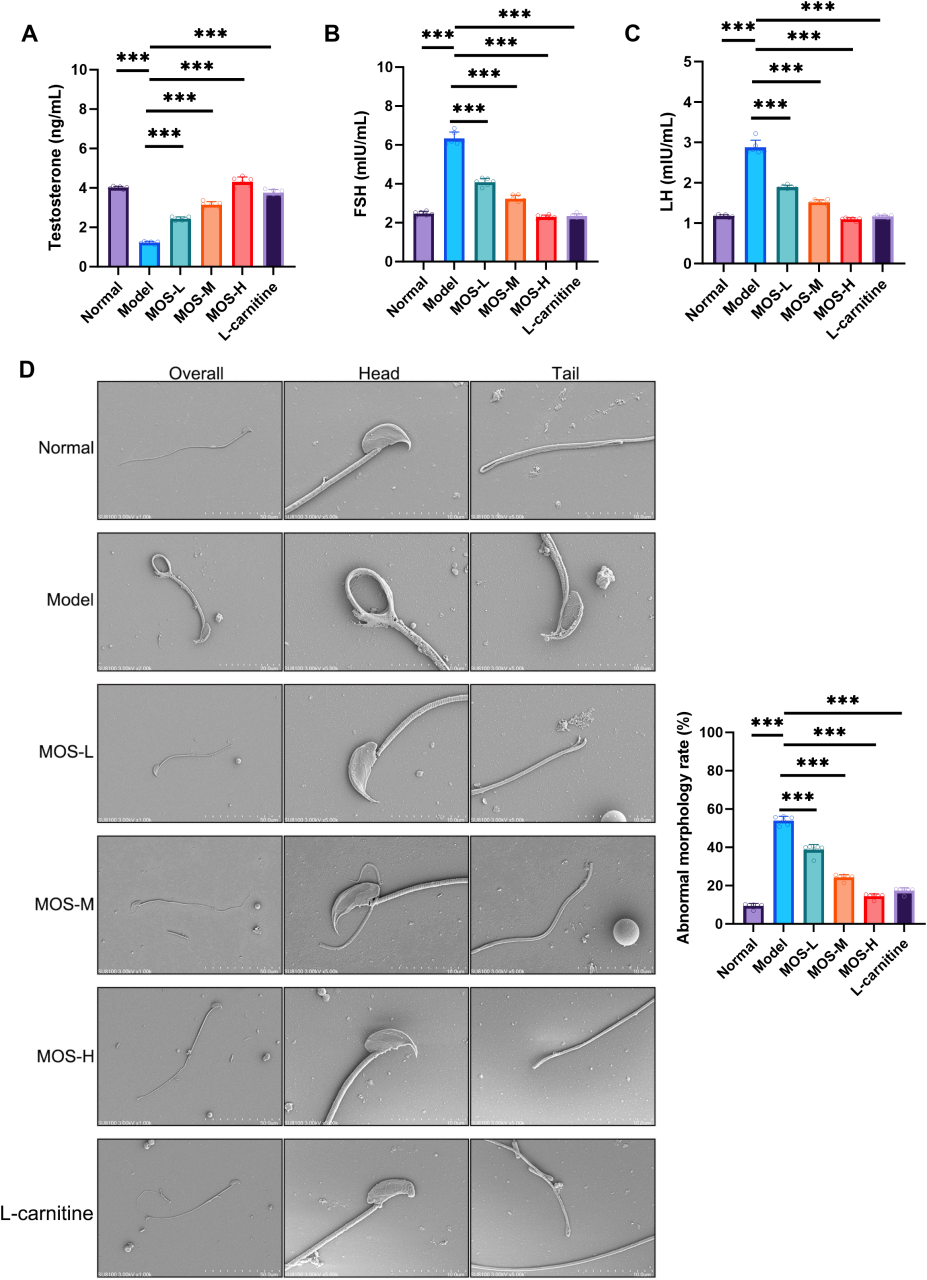
MOS improves sex hormone levels and reduces sperm morphological abnormalities in asthenozoospermic mice. **(A)** Serum testosterone (T) levels (*n* = 6). **(B)** Serum follicle-stimulating hormone (FSH) levels (*n* = 6). **(C)** Serum luteinizing hormone (LH) levels (*n* = 6). **(D)** Scanning electron microscopy of sperm morphology and statistical analysis of sperm morphological abnormalities (*n* = 6). Scale bar = 10 µm. Data are presented as mean ± SD. Compared with the model group, ^***^P < 0.001. MOS, *Morinda officinalis* oligosaccharides.

ELISA results revealed that serum testosterone (T) levels were significantly decreased in the Model group (**Figure 3A**), while FSH (**Figure 3B**) and LH (**Figure 3C**) were moderately elevated. MOS treatment restored T levels in a dose-dependent manner and reduced FSH and LH levels toward those of the Normal group. Similar trends were observed in the L-carnitine group, suggesting that MOS may partially restore hypothalamic–pituitary–gonadal axis function while improving sperm quality. Furthermore, both CASA analysis and scanning electron microscopy demonstrated a significant increase in abnormal sperm in the Model group, mainly characterized by head malformations and tail defects (**Figure 3D**). MOS treatment markedly reduced the proportion of abnormal sperm, with the greatest improvement observed in the high-dose group, and the L-carnitine group exhibited comparable effects. Collectively, these data indicate that MOS not only improves sex hormone profiles in asthenozoospermic mice but also reduces morphological abnormalities, thereby enhancing sperm quality.

### MOS restores gut microbiota diversity, promotes *Anaerococcus* abundance, and modulates related metabolic pathways

**Figure 4:**
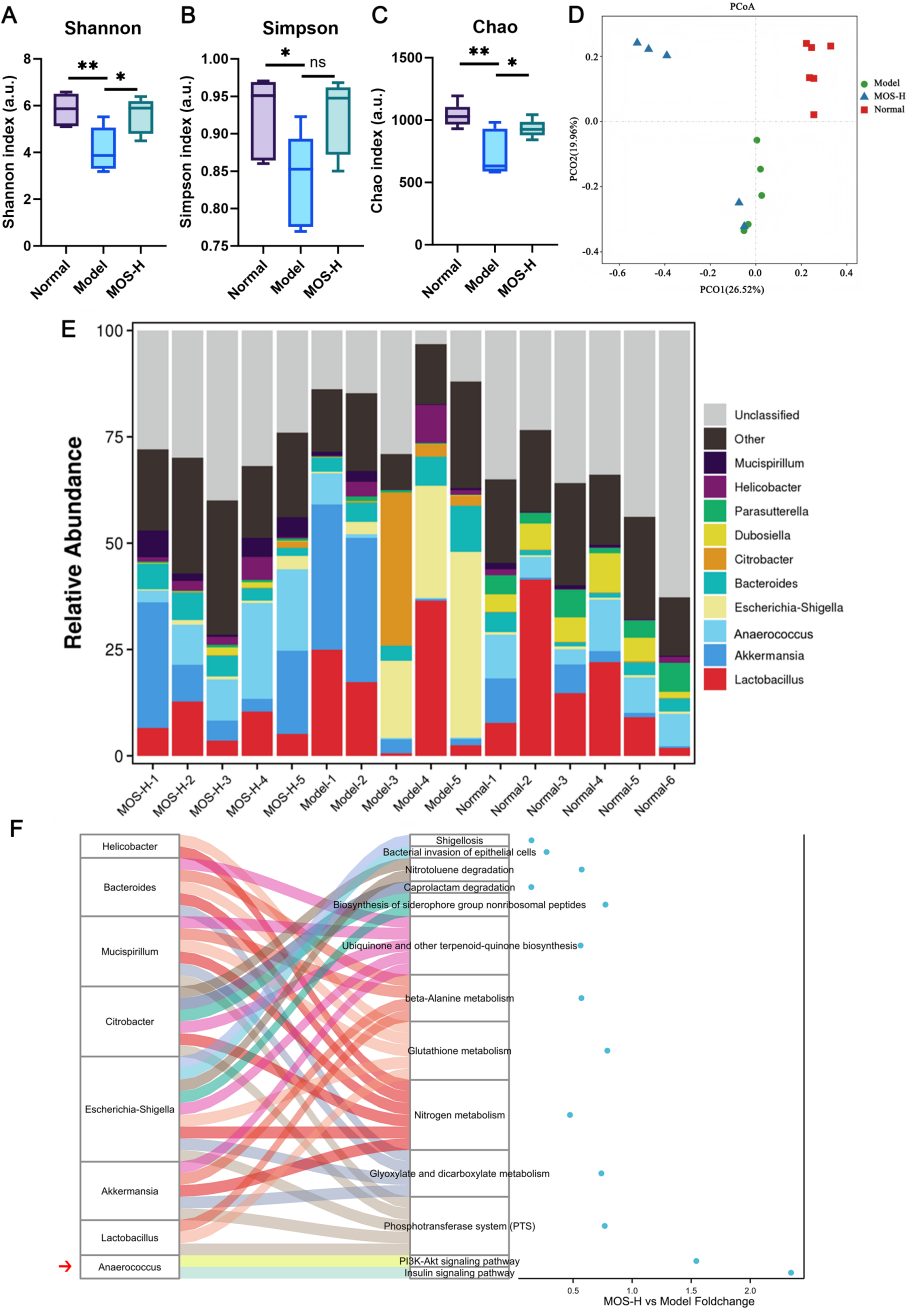
MOS improves gut microbiota diversity, promotes *Anaerococcus* abundance, and modulates related metabolic pathways in model animals. **(A)** Shannon index analysis of gut microbiota diversity. **(B)** Simpson index analysis of gut microbiota evenness. **(C)** Chao index analysis of gut microbiota richness. **(D)** PCoA analysis of gut microbiota structural differences. **(E)** Relative abundance of gut microbiota at the genus level. **(F)** Sankey diagram showing the associations between gut bacterial genera and predicted KEGG functional pathways based on PICRUSt2 analysis. Sample sizes were as follows: Normal (*n* = 6), Model (*n* = 5), and MOS-H (*n* = 5). Data are presented as mean ± SD. Compared with the model group, *P < 0.05, **P < 0.01, ns indicates no significant difference. PCoA, Principal coordinate analysis; KEGG, Kyoto Encyclopedia of Genes and Genomes; MOS, *Morinda officinalis* oligosaccharides.

Gut microbiota diversity was markedly reduced in the Model group, whereas high-dose MOS treatment significantly increased the Shannon index (**Figure 4A**), partially restored the Simpson index (**Figure 4B**), and significantly elevated the Chao index (**Figure 4C**). PCoA analysis revealed clear separation between the Normal and Model groups, while MOS treatment shifted microbial communities toward the Normal group profile (**Figure 4D**). At the genus level, *Lactobacillus* and *Escherichia-Shigella* abundances were increased in the Model group, whereas MOS intervention promoted *Anaerococcus* abundance and reshaped community composition (**Figure 4E**). Functional prediction further indicated multiple metabolic pathway differences between the Normal and Model groups. MOS treatment significantly affected amino acid metabolism, energy metabolism, and signaling pathways, potentially via *Anaerococcus*-mediated regulation of the PI3K–Akt and insulin signaling pathways (**Figure 4F**).

### MOS enhances IGF-1/PI3K/mTOR pathway activity and reduces apoptosis in model mice

**Figure 5:**
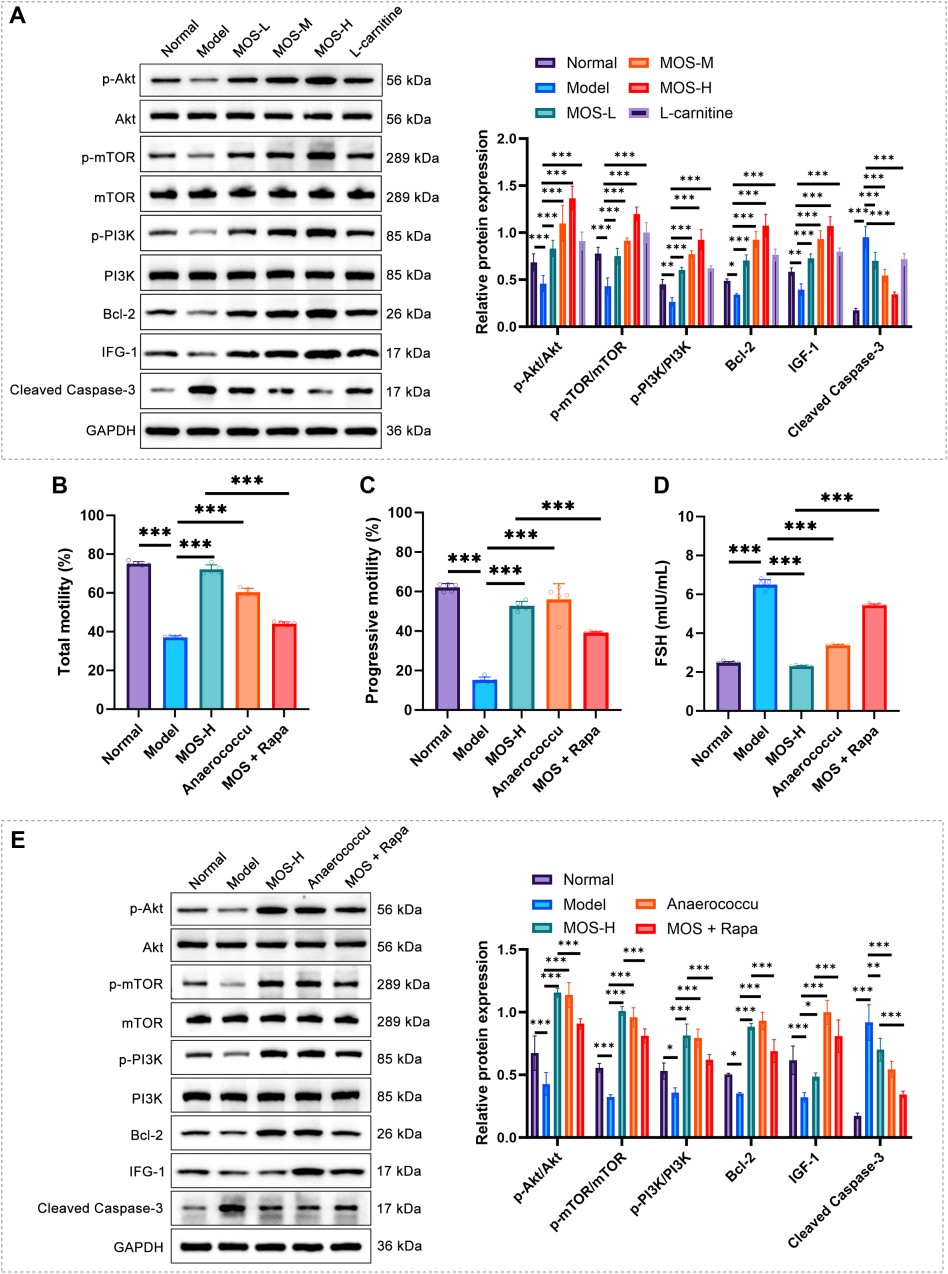
MOS improves sperm function by activating the IGF-1/PI3K/mTOR pathway. **(A)** Western blot analysis of protein expression of p-Akt, Akt, p-mTOR, mTOR, p-PI3K, PI3K, Bcl-2, IGF-1, and Cleaved Caspase-3 (*n* = 3). **(B)** Total sperm motility analysis (*n* = 5). **(C)** Progressive sperm motility analysis (*n* = 5). **(D)** Serum FSH levels detection (*n* = 5). **(E)** Western blot analysis of IGF-1/PI3K/mTOR pathway-related protein expression in different treatment groups (*n* = 3). Data are presented as mean ± SD. Compared with the model group, ^*^P < 0.05, ^**^P < 0.01, ^***^P < 0.001. Akt, Protein Kinase B; mTOR, Mammalian target of rapamycin; PI3K, Phosphoinositide 3-kinase; Bcl-2, B-cell lymphoma 2; IGF-1, Insulin-like growth factor 1; FSH, Follicle-stimulating hormone; Rapa, Rapamycin.

In the Model group, protein expression of p-mTOR/mTOR, p-Akt/Akt, p-PI3K/PI3K, Bcl-2, and IGF-1 was significantly reduced, whereas Cleaved Caspase-3 was increased. MOS treatment reversed these alterations in a dose-dependent manner (**Figure 5A**). Total and progressive sperm motility were significantly decreased in the Model group, but *Anaerococcus* transplantation markedly improved both parameters, similar to high-dose MOS treatment. Notably, MOS combined with rapamycin substantially attenuated the beneficial effects of MOS on sperm motility (**Figures 5B, C**). Serum FSH levels were significantly elevated in the Model group, while *Anaerococcus* transplantation and MOS treatment both reduced FSH, an effect partially reversed by rapamycin co-treatment (**Figure 5D**). Western blot analyses further demonstrated that *Anaerococcus* transplantation enhanced IGF-1/PI3K/mTOR signaling and reduced apoptosis, whereas rapamycin markedly weakened MOS-mediated pathway activation, thereby partially abrogating its protective effects (**Figure 5E**).

## Discussion

Using a gossypol-induced asthenozoospermia mouse model, this study demonstrated that MOS improved total and progressive sperm motility, normalized reproductive hormones, and suppressed germ cell apoptosis. Gossypol is a polyphenolic compound known to impair sperm mitochondrial function, suppress ATP production, and disrupt spermatogenesis through oxidative injury and energy deprivation (25). Prior animal studies showed that gossypol exposure leads to significant reductions in sperm motility, serum testosterone, and testicular histology, typically achieved by 15–20 mg/kg oral dosing over 3–4 weeks (22, 26, 27). Although oxidative stress markers were not directly assessed in the present study, previous findings have indicated that MOS alleviates reproductive injury via antioxidant and mitochondrial protective effects (20, 28). Consistent with previous reports, gossypol exposure markedly reduced sperm concentration and motility in the present study, accompanied by decreased luminal sperm content and prominent vacuolation in the epididymis, reflecting disrupted sperm maturation and transport (27). While severe seminiferous epithelial degeneration and renal mesangial alterations have been observed under higher-dose or continuous gossypol exposure (29), the current regimen did not produce overt structural lesions in the testes, liver, or kidneys. Nonetheless, the observed reduction in white blood cell counts and elevated BUN in the Model group align with reports that gossypol can perturb hematological homeostasis and induce subclinical nephrotoxicity (30). MOS supplementation effectively restored sperm motility parameters and normalized WBC and BUN levels, indicating both reproductive protection and favorable systemic tolerance. It is worth noting that although WBC counts differed significantly among groups, all values remained within the physiological range and likely reflect normal physiological fluctuation. These findings not only support the notion that gossypol primarily targets reproductive tissues but also highlight the potential of MOS to counteract gossypol-induced functional and hematological disturbances while maintaining a favorable safety profile.

MOS comprises a structurally diverse mixture of oligosaccharides, including bajijiasu, sucrose, 1-kestose, nystose, fructofuranosylnystose, and various inulin-type hexasaccharides to nonasaccharides (18, 31). Due to their large molecular size and high polarity, most MOS components exhibit low intestinal permeability and are unlikely to be absorbed in intact form (20). Their circulating or tissue concentrations are expected to be negligible. Therefore, the biological effects observed in this study are likely mediated through microbial fermentation products such as SCFAs rather than direct systemic absorption of MOS. However, certain low-molecular-weight components such as bajijiasu have demonstrated direct bioactivity following oral administration, including modulation of hormone levels, enhancement of sperm quality, and antioxidant effects (32). These data suggest that the therapeutic effects of MOS may involve a dual mechanism, including direct actions from systemically absorbed constituents like bajijiasu, and indirect effects mediated through gut microbiota remodeling. This interpretation is consistent with recent findings in CUMS (chronic unpredictable mild stress) mouse models, where MOS restored sexual motivation, normalized serum sex hormones, corrected epididymal sperm defects, and reshaped gut microbiota (20).

The present results further support the involvement of the gut–testis axis in male reproductive health. MOS treatment markedly increased the abundance of *Anaerococcus*, an SCFA-producing genus that contributes to mucosal homeostasis through butyrate generation (33–36). Additionally, MOS restored α-diversity (Shannon and Chao indices), reduced the abnormal overrepresentation of *Escherichia-Shigella* and *Lactobacillus* observed in the model group, and shifted overall microbial composition toward the healthy phenotype. These results align with prior reports showing that asthenozoospermic patients exhibit decreased gut microbial diversity and dysbiosis marked by increased abundance of *Bacteroides*, *Prevotellaceae*, and *Erysipelatoclostridium* (37–39). Since fecal SCFAs were not quantified and other butyrate-producing taxa (e.g., *Bacteroides*, *Clostridium*) were not systematically evaluated, this remains a limitation that warrants future investigation. Targeted metabolomic profiling and 16S-based functional predictions will help clarify which microbial functions are most closely tied to sperm improvement.

A central mechanistic finding of this study is the activation of the IGF-1/PI3K/mTOR signaling cascade following MOS and *Anaerococcus* intervention. IGF-1, acting via IGF-1R, activates PI3K/Akt/mTOR to promote protein synthesis, inhibit autophagy, and support cellular survival—processes critical for spermatogonial stem cell self-renewal, Sertoli cell function, and overall spermatogenesis (40–42). Disruption of this axis impairs cytoskeletal remodeling during spermiation and leads to defective sperm release (43, 44). Moreover, PI3K/Akt-dependent control of actin-binding proteins such as LCRMP-1 is required for cytoskeletal remodeling during spermiation, and disruption of this pathway leads to defective sperm release and subfertility (45). In this study, MOS activated the IGF-1/PI3K/mTOR axis and improved progressive motility, in line with evidence that rescuing Akt/mTOR signaling (e.g., in ornidazole-induced asthenozoospermia) improves sperm quality partly by curbing excessive autophagy (46). MOS administration increased IGF-1 expression, enhanced phosphorylation of PI3K, Akt, and mTOR, and upregulated Bcl-2 while suppressing Cleaved Caspase-3, indicating reduced germ cell apoptosis. These molecular changes coincided with improved sperm motility. Rapamycin, a specific mTOR inhibitor, attenuated these effects, supporting a causal link between IGF-1 pathway activation and reproductive improvement. Functional validation through *Anaerococcus* transplantation reproduced these key effects, suggesting that *Anaerococcus* enrichment partially recapitulates MOS-induced activation of the IGF-1/PI3K/mTOR axis and sperm improvement in this model. This microbiota–signaling linkage represents a novel mechanistic insight that provides a biological basis for MOS-induced reproductive recovery.

Despite these findings, several limitations must be acknowledged. First, all data were obtained in a murine model, human applicability remains to be confirmed through clinical validation. Second, oxidative parameters (ATP, ΔΨm, ROS), mitochondrial function, and downstream mTOR targets (p70S6K, 4E-BP1) were not assessed, which limits mechanistic granularity. Third, although *Anaerococcus* transplantation provided genus-specific evidence for microbiota involvement, future studies should include transplantation of a non-specific gut bacterial strain (e.g., *Lactobacillus plantarum*) as an additional control to disentangle genus-specific from general microbial effects. Lastly, although our data indicate that *Anaerococcus* enrichment and IGF-1/PI3K/mTOR pathway activation occur in parallel with improved sperm function, direct biochemical validation is warranted to confirm mechanistic links and to explore contributions from other SCFA-producing taxa. Future studies incorporating multi-omics tools and gnotobiotic models will be essential to fully define the microbiota-dependent and microbiota-independent contributions of MOS to reproductive health.

## Conclusion

**Figure 6:**
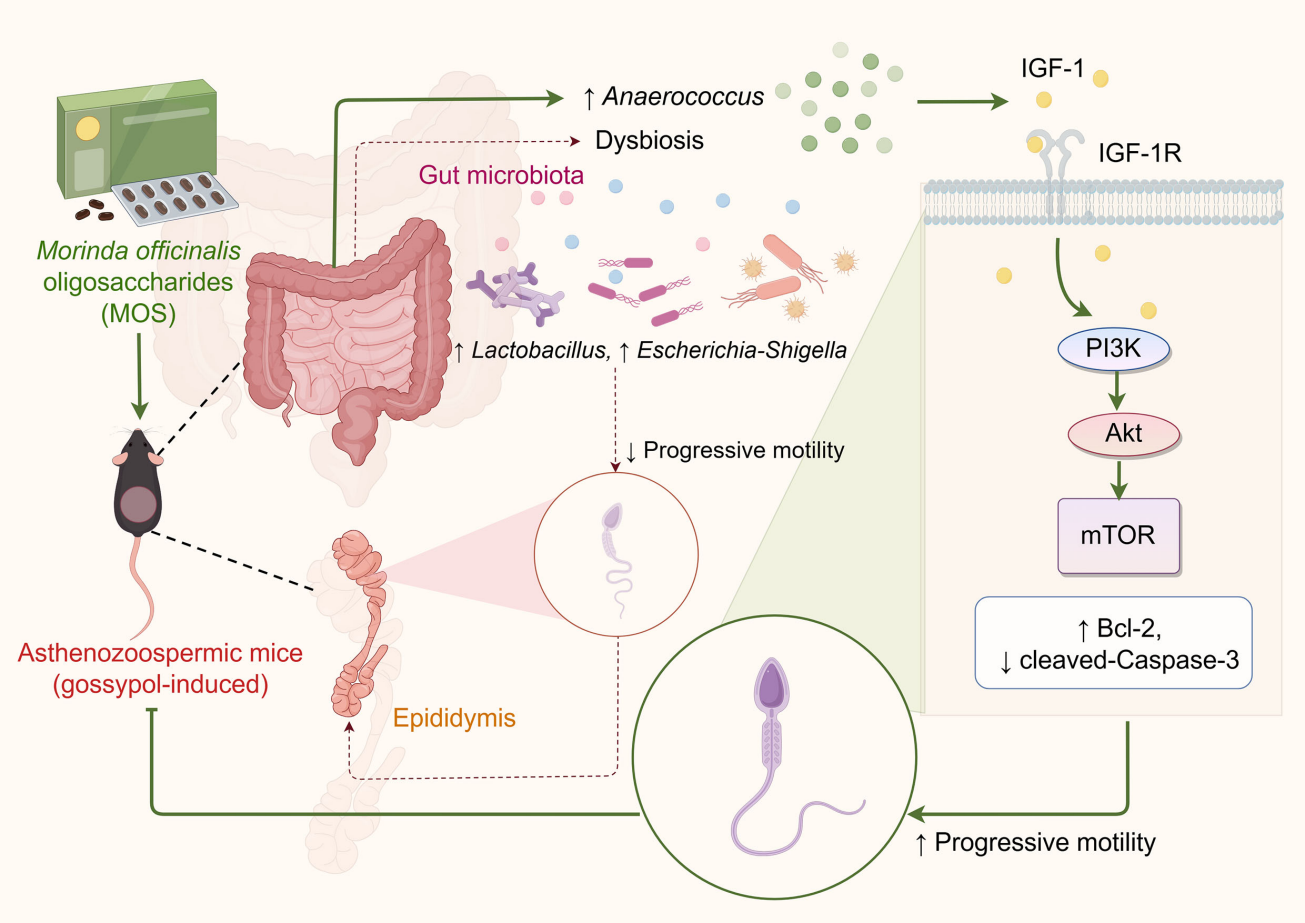
Proposed mechanism by which MOS improves sperm motility via gut microbiota modulation and activation of the IGF-1/PI3K/mTOR signaling pathway.

In a gossypol-induced asthenozoospermia model, MOS significantly improved sperm motility and reduced morphological abnormalities with a favorable safety profile. Mechanistically, MOS appears to remodel the gut microbiota, including enrichment of *Anaerococcus*, and is associated with activation of the IGF-1/PI3K/mTOR pathway in parallel with sperm functional recovery (**Figure 6**).

## Data Availability

The raw data supporting the conclusions of this article will be made available by the authors, without undue reservation.
